# Mucin 1 Gene (*MUC1*) and Gastric-Cancer Susceptibility

**DOI:** 10.3390/ijms15057958

**Published:** 2014-05-07

**Authors:** Norihisa Saeki, Hiromi Sakamoto, Teruhiko Yoshida

**Affiliations:** Division of Genetics, National Cancer Center Research Institute, Tsukiji 5-1-1, Chuo-ku, Tokyo 104-0045, Japan; E-Mails: hsakamot@ncc.go.jp (H.S.); tyoshida@ncc.go.jp (T.Y.)

**Keywords:** gastric cancer, mucin 1, *Helicobacter pylori*, genome-wide association study, single nucleotide polymorphism, cancer susceptibility gene

## Abstract

Gastric cancer (GC) is one of the major malignant diseases worldwide, especially in Asia. It is classified into intestinal and diffuse types. While the intestinal-type GC (IGC) is almost certainly caused by *Helicobacter pylori* (HP) infection, its role in the diffuse-type GC (DGC) appears limited. Recently, genome-wide association studies (GWAS) on Japanese and Chinese populations identified chromosome 1q22 as a GC susceptibility locus which harbors mucin 1 gene (*MUC1*) encoding a cell membrane-bound mucin protein. *MUC1* has been known as an oncogene with an anti-apoptotic function in cancer cells; however, in normal gastric mucosa, it is anticipated that the mucin 1 protein has a role in protecting gastric epithelial cells from a variety of external insults which cause inflammation and carcinogenesis. HP infection is the most definite insult leading to GC, and a protective function of mucin 1 protein has been suggested by studies on *Muc1* knocked-out mice.

## 1. Introduction

Gastric cancer (GC) is one of the major cancers and the second most deadly form of cancer worldwide [1]. Gastric adenocarinoma, a major type of GC, can be histologically classified into two types: intestinal and diffuse, a classification that is thought to reflect its pathogenesis [2]. In the carcinogenesis of the intestinal-type GC (IGC), *Helicobacter pylori* (HP) infection has an important role. The HP infection results in a sequence of inflammatory change of the gastric epithelium leading to neoplasma: chronic inflammation-intestinal metaplasia-dysplasia-adenocarcinoma [3]. On the other hand, diffuse-type GC (DGC) is thought to develop as a consequence of some genetic change that occurred in gastric stem cells and/or epithelial precursor cells.

In the carcinogenic contribution by HP infection, two bacterial proteins are important: CagA, a product of the cytotoxin-associated gene A, and vacuolating cytotoxin (VacA). There are many excellent review articles on the function of the two proteins. In brief, the proteins induce signaling related to the pro-inflammatory (e.g., interleukin-17, -21 and Nod1), proliferative (e.g., epidermal growth factor-related peptides, EGF receptor, Ras-MAPK pathway, cyclooxygenase 2 and nuclear translocation of β-catenin) and anti-apoptotic (e.g., nuclear factor kappa-B) pathways in the gastric epithelial cells [4].

Consequently, the likelihood is that almost all IGC can be prevented by the eradication of HP infection, and the International Agency of Research on Cancer, sponsored by the World Health Organization, has categorized HP as a class I carcinogen and a definite cause of human gastric cancer, contributing to about 75% of the cases [4,5]. Although the contribution of HP infection is suggested [6], DGC has no established environmental risk factor but does have a tendency to develop in younger people than does IGC, suggesting a genetic factor as a major contributor in its carcinogenesis. Moreover, some countries have a higher prevalence of HP infection but a much lower GC incidence than other countries. Japan, for example, is a country with a high incidence of GC (age-standardized incidence rate 62.7/100,000) but a lower HP seroprevalence (39.3%) than other Asian countries such as Bangladesh (92%) and India (79%), which have a much lower GC incidence, 1.6/100,000 and 5.7/100,000, respectively [7]. The geographical enigma suggests that genetic factors may also contribute to IGC development. With this as a background, three genome-wide association studies (GWASes) were recently performed for detecting the genetic factors related to GC susceptibility, and two of them identified chromosome 1q22 harboring the mucin 1 (*MUC1*) gene as a GC susceptibility locus [8–11].

## 2. Association between GC (Gastric Cancer) and *MUC1*

A common disease-common variant hypothesis proposes the idea that common and multifactorial diseases are attributed by multiple common genetic variants with a weak to moderate pathogenic effect [12]. Single nucleotide polymorphisms (SNPs) are genetic variants and observed on average once in every 300 nucleotides, which means there are roughly 10 million SNPs in the human genome. Although the Mendelian inheritance law states that separate genetic loci are passed independently of one another from parents to offspring, the SNPs actually descend to offspring as multiple clusters, *i.e.*, many SNPs linked to each other in each chromosome, because there are recombination hotspots in each chromosome when crossing-over events occur during mitosis. This condition, *i.e.*, the SNPs existing as heritable clusters rather than conforming to the Mendelian inheritance law in the genome is called linkage disequilibrium (LD), and current GWASes using SNPs have been exploring genetic susceptibility loci using LD in the genome [13]. In GWASes, an association of SNPs with a disease suggests that the genetic factors or genes exist in the clusters (called LD block or haplotype block) to which the SNPs belong.

In general, each ethnic population has a distinct set of SNPs and haplotypes. In Japan, the SNPs were already catalogued in the early 2000s as a JSNP (Japanese SNP) database [14,15]. The database contributed to a number of GWASes on genetic factors for common diseases including, for example, lung cancer, myocardial infarction, asthma, intracranial aneurism and Kawasaki disease [16–20].

Japan is a country with one of the highest GC incidences, *i.e.*, it is a common disease in the population. Recently, we performed a GWAS on DGC, which consisted of two steps of the association study [8]. The first step was performed on 85,576 SNPs using 188 DGC cases and 752 references, and the second step on 2753 selected SNPs with 749 DGC cases and 750 controls. Finally, we identified ten SNPs related to DGC with statistical significance, which included four SNPs located in chromosome 8q24.3 and 2 SNPs in 1q22. Haplotype block analyses for detecting the susceptibility genes revealed two candidates at 8q24.3 and 5 at 1q22.

In the 8q24.3 haplotype block, prostate stem cell antigen gene (*PSCA*) was identified as a DGC susceptibility gene, with a significant association between DGC and two SNPs in the gene (rs2976392: 926 cases, 1397 controls, allele-specific odds ratio = 1.71, 95% confidence interval = 1.50–1.94, *p* = 1.5 × 10^−16^; rs2294008: 925 cases, 1396 controls, allele-specific odds ratio = 1.67, 95% confidence interval = 1.47–1.90, *p* = 2.2 × 10^−15^) [8]. The association was replicated in the Korean population, which has a GC incidence as high as the Japanese (rs2976392: 449 cases, 390 controls, allele-specific odds ratio = 1.90, 95% confidence interval = 1.56–2.33, *p* = 8.0 × 10^−11^; rs2294008: 454 cases, 390 controls, allele-specific odds ratio = 1.91, 95% confidence interval = 1.57–2.33, *p* = 6.3 × 10^−11^) [8]. *PSCA* also showed a weak correlation to IGC in populations from both Japan (rs2976392: 599 cases, 1397 controls, allele-specific odds ratio = 1.29, 95% confidence interval = 1.12–1.49, *p* = 5.0 × 10^−4^) and Korea (rs2976392: 416 cases, 390 controls, allele-specific odds ratio = 1.37, 95% confidence interval = 1.12–1.68, *p* = 0.0017) [8]. Later, the association of rs2976392 or rs2294008 with GC was validated in other Japanese and Korean panels and also in Chinese and Caucasian populations [21–28].

**Figure 1.**
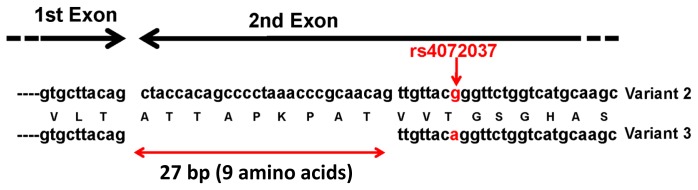
SNP (single nucleotide polymorphism) rs4072037 (G/A, red arrow) in the *MUC1* gene determines the major splicing variants expressed in the gastric mucosa. In the gastric mucosa, major splicing forms were variants 2 and 3, and the allele of SNP rs4072037 is related to the splicing acceptor site selection in the second exon (1st and 2nd exons are indicated by black arrows) and consequently determines the variant type. The variant 2 but not the variant 3 transcript contains the first 27 bp (double-headed red arrow) of the 2nd exon.

In the other DGC susceptibility locus 1q22, the haplotype block contained five genes, in which we identified the mucin 1 gene (*MUC1*) as the susceptibility gene [9]. A representative SNP in *MUC1*, rs2070803, showed the association with DGC (*p* = 2.20 × 10^−6^, adjusted per allele OR = 1.63, 606 cases and 1264 controls), which was replicated in additional Japanese (*p* = 3.93 × 10^−5^, OR = 1.81, 304 cases and 1465 controls) and Korean (*p* = 2.19 × 10^−4^, OR = 1.82, 452 cases and 372 controls) case-control panels. Moreover, we identified a functional SNP rs4072037 (A/G) in the *MUC1* gene, and the A allele was associated with DGC patients [9]. The SNP influences the splicing of the primary transcripts. We revealed that there are two major *MUC1* transcripts in the gastric epithelium: variants 2 and 3. The rs4072037 located in the 5′ side of the second exon determines the splicing acceptor site in the second exon, which in turn determines the type of variants; the G and A alleles result in the expression of variants 2 and 3, respectively (Figure 1) [9,29]. The structural difference between the two variants is nine amino acids in the second exon that are involved in the *N*-terminal signal peptide. This difference in the signal peptide may lead to a difference in the function of the encoded protein between the two splicing variants.

In addition to the GWAS conducted in Japan [8,9], GWASes on other ethnic populations also listed 1q22 as a candidate for a GC-related locus (Table 1). The GWAS on the Chinese population revealed the association between the rs4072037 in *MUC1* and GC (rs4072037; OR = 0.75, *p* = 4.22 × 10^−7^) [11].

Besides the GWAS, the association of SNPs in *MUC1* with GC has been demonstrated in other ethnic populations, especially in Chinese (Table 1). An association study with imputation analysis on Chinese case-control samples demonstrated the association of the SNP (OR = 0.73, *p* = 1.0 × 10^−4^) [10]. In a study on 300 cases and 300 controls, the association was also successfully replicated (rs2070803 AA/AG to GG, OR = 0.46, the permutation *p* < 0.001) [32]. Still another study on the Chinese (138 cases and 241 controls) showed the association (rs4072037 AA against AG + GG, OR = 1.81, 95% CI = 1.06–3.12) [31]. In addition to the Chinese populations, the association was also replicated in the Korean population (3245 cases and 1700 controls, rs4072037 AG to AA, OR = 0.78, 95% CI = 0.67–0.91) [30]. Moreover, it was replicated in a study on a Caucasian population (290 cases and 376 controls) in which an association between rs4072037 and non-cardia intestinal GC was demonstrated (OR = 0.4, 95% CI = 0.2–0.9) [34]. Another study on 273 cases and 377 controls also revealed the association (rs4072037 AA against GG, OR = 2.20, the permutation *p* < 0.01) [33]. Finally, a meta-analysis on the data obtained in the association studies with Asian or European ethnicities showed an association of rs4072037 with both IGC (G allele, OR = 0.74, 95% CI = 0.66–0.83, *p* value of *Z*-test = 1.79 × 10^−7^) and DGC (G allele, OR = 0.66, 95% CI = 0.58–0.74, *p* value of *Z*-test = 1.29 × 10^−7^) [35]. It is noteworthy that the A allele was associated with GC and is a major allele in the Japanese, Chinese and Korean populations, which have a high GC incidence, but a minor one in a European population with a low GC incidence.

Surprisingly, an association between *MUC1* gene polymorphisms other than SNP and GC has also been demonstrated in other studies previous to the GWASes. The *MUC1* gene has a variable tandem repeat region, which results in large (L) and small (S) alleles shown in Southern blot analyses when DNA samples are digested with restriction enzymes. It was demonstrated in a Caucasian population (159 GC cases and 324 controls) that SS genotypes of *MUC1* had an increased risk of developing GC (SS to LL, OR = 4.3, 95% CI = 1.8–10.5, *p* < 0.0001) [36], and the two alleles, the S and the A of rs4072037, as well as the L and the G of the SNP are in LD, respectively, in Japanese and European populations [9,29]. The association in different ethnic populations strongly supports the suggestion that *MUC1* is a GC susceptibility gene.

## 3. MUC1 Expression in Gastric Carcinogenesis

Several immunohistochemical studies identified the mucin 1 protein in normal and malignant gastric epithelial cells. However, the pattern of the staining for the protein was a little different depending on the antibodies used in the studies, which is likely to have originated from variableness in the glycosylation state of the antigen used in raising the antibodies. In summary, the MUC1 protein was observed in the surface foveolar cells in the entire stomach, in mucous neck cells and chief cells of the gastric fundus and antrum, and also in the pyloric gland, typically in the manner of staining at the apical side of the cell membrane and also diffusely in cytoplasm [37–39]. An immunohistochemical study using two anti-mucin 1 antibodies, HMFG1 reacting with the fully glycosyrated mucin 1 protein and SM3 reacting with the under-glycosylated protein, revealed a zonal pattern of the glycosylation state of the protein [40]. The HMFG1 stained the protein in the foveolar cells of the antrum but not of the corpus. On the other hand, staining of SM3 was limited to the perinuclear area of the foveolar cells of the antrum.

There are many immunohistochemical studies on MUC1 expression in GC, and most of them reported MUC1 staining in roughly more than 50% of both IGC and DGC except for signet-ring cell carcinoma, a poorly differentiated GC in which the MUC1 expression was observed only 10% (Table 2) [38–46]. Although MUC1 staining seems to be related to a better differentiation state of the tumor cells since it can be considered as a differentiation marker, most of the reports suggested an association of MUC1 expression with a worse prognosis. It was also reported that abnormal E-cadherin expression in tumor cells was correlated to MUC1 expression, which was observed in the cases of poor prognosis or advanced stage [47,48]. Downregulation of MUC1 was observed in pre-cancerous lesion. There are two types of intestinal metaplasia, complete and incomplete: the former has fully developed intestinal goblet cells and enterocytes with a brush border and the latter has no absorptive cells [49]. Several studies revealed none, or a marked reduction of MUC1 expression in the tissues of complete intestinal metaplasia, a pre-neoplastic condition, although it was expressed in the incomplete type [37,39,40,50,51]. The suppression in the pre-neoplastic lesion and the frequent reactivation in GC of MUC1 expression, especially in the cases with a poor prognosis, implicated its distinct function in normal gastric epithelial cells and in GC cells.

The structure and function of the promoter region of *MUC1* gene have been elucidated. It contains responsive elements for several signalings executed by external molecules, such as transforming growth factor-β and interferon-γ. Moreover, hypomethylation of the tandem-repeat region is required for *MUC1* gene expression in epithelial tissues [52].

## 4. MUC1 Function in Normal Gastric Epithelial Cells

MUC1 belongs to the mucin family (MUC1 to MUC21), which consists of secretory and membrane-bound types, and MUC1 is the latter [53]. In normal epithelial cells, MUC1 is located at the apical surface of the cells and acts as a barrier against exogenous insults to the cells [54]. The MUC1 protein on the cell surface consists of *N*- and *C*-terminal subunits, designated as MUC1-N and MUC1-C, respectively. After being translated, a single MUC1 protein is cleaved to the two subunits by autoproteolysis, but both the subunits remain associated by non-covalent binding and are localized to the cell membrane. MUC1-N, present on the cell surface, has multiple glycosylation sites and has a protective role for cells against many types of insults [55].

HP infection is a definite carcinogen for gastric epithelial cells, leading to carcinogenesis, and there is experimental and epidemiological evidence for the role of MUC1 in protecting the gastrointestinal tract from bacterial infection. *Muc1* knocked-out (KO) mice with oral infection of *Campylobactor jejuni*, showed damage in the small intestine as well as systemic infection more frequently than did the wild type [56]. A study on *Muc1*-deficient cultured cells and mice demonstrated that mucin 1 protected the gastric epithelium from both non-MUC1 binding bacteria (by inhibiting adhesion to the cell surface with its steric hindrance effect) and MUC1-binding bacteria (by acting as a releasable decoy) [57]. In one study, mice lacking *Muc1* were colonized by five-fold more HP within one day of infection, and developed an atrophic gastritis marked by loss of parietal cells, although wild-type mice developed only a mild gastritis, when infected for two months with HP [58].

As mentioned previously, our study demonstrated that rs4072037 determines a major variant expressed in the stomach by influencing the splicing acceptor site of the second exon (Figure 1) [9]. It is likely that rs4072037 affects the barrier function in the stomach of individuals through this determination of a major variant. In addition, our study revealed that rs4072037 also influences the transcriptional activity of the *MUC1* gene promoter; the A allele associated with GC reduced the transcriptional activity, which may result in decreased MUC1 expression [9]. These findings suggest that rs4072037 influences the quantity and/or the quality of the MUC1 protein, which causes a difference in its barrier function in the stomach and subsequently the difference in GC susceptibility between individuals. Indeed, it was reported on Caucasians that those having the S allele of *MUC1*, which is linked to the A allele of rs4072037, were more susceptible to HP gastritis than the people with the L allele [59]. A study on a Chinese population revealed that HP seropositivity and AA genotypes for rs4072037 synergistically enhance the risk of GC [60]. In the study, compared to the subjects with HP seronegativity and the AG or GG genotype, those with HP seropositivity and the AG or GG genotype had more risk (OR = 2.30, 95% CI = 1.23–4.31, *p* = 0.017), and those with HP seropositivity and the AA genotype has significant risk (OR = 3.95, 95% CI = 2.29–6.79, *p* = 6.5 × 10^−6^). However, as the risk of those with HP seronegativity and the AA genotype was also increased (OR = 2.46, 95% CI = 1.42–4.27, *p* = 0.003), it is certain that the genotype would also contribute to GC development in an HP-independent manner. The effect of HP seropositivity and rs4072037 state is summarized in Table 3 [9,59,60].

Besides the protective function as a mucosal barrier, MUC1 may have an anti-carcinogenic role in another manner. As previously mentioned, the MUC1 protein consists of *N*- and *C*-terminal subunits, MUC1-N and MUC1-C. MUC1-C has a transmembrane domain and a cytoplasmic tail (CT), which contains several phosphorylation sites and a β-catenin binding site. Phosphorylation of threonine contained in the CT promotes interactions between MUC1 and β-catenin, and leads to a nuclear localization of the complex, resulting in regulation of genes including *p53* [61,62]. Namely, the CT is involved in subcellular signal transduction. Recently it has been suggested that the HP virulence factor CagA destabilizes the E-cadherin/β-catenin complex located in the cytoplasm of epithelial cells and enhances an accumulation of β-catenin in the nucleus [63]. The nuclear accumulation of β-catenin activates beta-catenin-dependent genes, such as *CDX1*, which encodes an intestinal specific transcription factor, and induces aberrant expression of molecules in gastric epithelial cells, including an intestinal-differentiation marker, goblet-cell mucin MUC2, which contributes to the development of intestinal metaplasia, a pre-neoplastic lesion [64]. In addition, the nuclear accumulation of β-catenin also activates interlukin-8 expression, a chemotactic and inflammatory cytokine [65]. It is hypothesized that MUC1 binds to β-catenin and attenuates its nuclear accumulation [66,67]. Intriguingly, it was demonstrated that HP upregulates MUC1 expression in gastric cancer cells through STAT3 and CpG hypomethylation [68]. This cascade may exist in the normal gastric epithelium as an anti-carcinogenic mechanism against HP infection. It was reported that HP infection upregulates MUC2, MUC5AC and MUC6 genes in KATO-III, a cultured gastric cancer cell line [69]; however, it was demonstrated that HP infection reduced the rate of mucin turnover and decreased the levels of Muc1 in the gastric mucosa of mice [70].

## 5. MUC1 Function in Gastric Carcinogenesis

Contrary to its protective function in normal gastric epithelial cells, the two findings mentioned above suggest a different function of MUC1 in GC cells: the gene is silenced in intestinal metaplasia, a pre-neoplastic lesion, but frequently reactivated in GC, and its expression is correlated to poor prognosis. Indeed, MUC1 has been considered as an oncoprotein, because there is accumulating evidence which suggests its cancer-promoting function.

It was reported that, interacted with Kruppel-like factor 4 (KLF4), a MUC1 *C*-terminal subunit (MUC1-C) occupies the PE21 element of the *p53* gene promoter, which recruits histone deacetylases, and suppresses the transcription of the *p53* gene [71]. *p53* is one of the representative tumor suppressor genes functioning in apoptosis, genomic stability and the inhibition of angiogenesis. It is a master guardian and executioner that surveys genetic damage and responds to it by arresting the cell cycle and facilitating DNA damage repair, or by induction of cell death when the genetic damage is severe [72]. MUC1 activates anti-apoptotic protein Bcl-xL and attenuates the loss of mitochondrial transmembrane potential, mitochondrial cytochrome c release and caspase-9 activation, leading to the failure of apoptosis induction [73]. In response to DNA damage, the non-receptor c-Abl tyrosine kinase is translocated to the nucleus and induces apoptosis of the cells, but MUC1 protein attenuates this nuclear translocation [74]. As stated above, MUC1 is a tremendous oncoprotein that destroys apoptosis execution pathways, one of the most important anti-cancer machines contained in the cells. The anti-apoptotic function of molecules confers cancer cells with resistance to genotoxic anticancer drugs.

MUC1 may contribute to metastasis, as it was demonstrated *in vitro* that the MUC1 protein can bind to intercellular adhesion molecule-1 (ICAM-1), which facilitates adhesion of breast cancer cells to endothelial cells, leading to adhesion and subsequent migration through the vessel wall [75].

Moreover, MUC1 could have some role in GC stem cells, as it acts as a growth factor receptor on undifferentiated human embryonic stem cells and is expressed in acute myeloid leukemia stem cells [76,77]. Intriguingly, it is also known that MUC1 facilitates cancer cell survival under hypoxic and nutrient-deprived conditions by regulating glucose and lipid metabolism and the cellular energy state [78].

As previously mentioned, in normal epithelial cells, it is likely that the G allele of rs4072037 contributes to increasing MUC1 expression and maybe also to enhancing the quality of MUC1 protein. It would be interesting to know whether the G allele is correlated to a poor prognosis in GC, but no study on the relation of the SNP and a GC prognosis has yet been conducted.

## 6. Perspective and Conclusions

**Figure 2.**
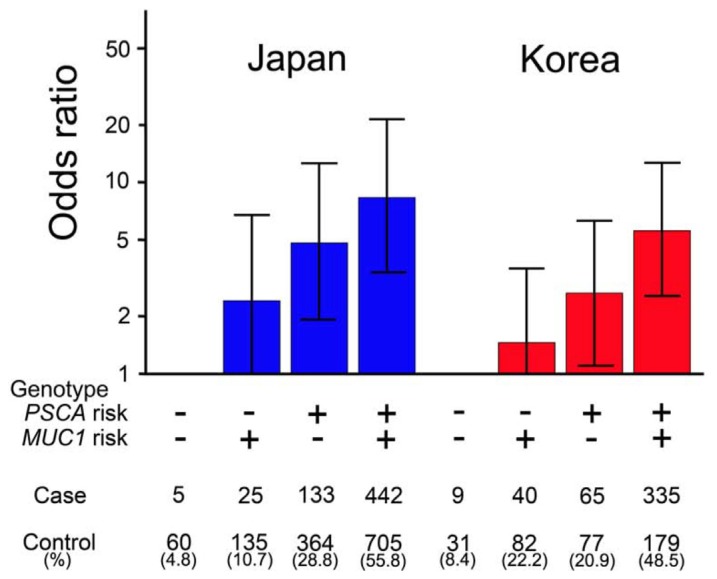
Japanese and Korean populations can be stratified based on *PSCA* and *MUC1* genotypes associated with risk for DGC. The stratification and risk estimation were performed using genotype data of rs2294008 in *PSCA* and rs4072037 in *MUC1* [9]. The risk allele’s effect is assumed to be dominant for rs2294008 in *PSCA* (risk genotype: TT and TC; protective genotype: CC) and recessive for rs4072037 in *MUC1* (risk genotype: AA; protective genotype: GG and GA). Bar, upper and lower bounds of 95% confidence interval.

Needless to say, prevention is the best way for us to cope with diseases. GC susceptibility genes, which have been and will continue to be identified, may contribute to GC prevention because a population can be stratified based on the GC susceptibility defined by the genes. The stratification enables us to intervene in the subpopulations by, for example, modulating the intensity of health check procedures according to their GC-development risk: health check with an endoscopic examination every two years starting at age 40 or with an examination every six months starting at age 20. Interestingly, the Japanese and Korean populations can be stratified using the two GC susceptibility genes (Figure 2). The combined genotype association data of rs2294008 in *PSCA* and rs4072037 in *MUC1* suggested that 4.8% of the Japanese population has the risk genotype of rs4072037, 28.8% the risk genotype of rs2294008 and 55.8% with both, and that the people of the double risk genotype has the highest risk for GC development (OR = 8.4) [9,79]. Unfortunately, the DNA samples used in this study were not linked with the information on HP infection, but it is likely that the highest risk group can be further stratified based on HP infection state and other environmental factors. The combination of HP detection and the identification of the rs4072037 and rs2294008 genotypes may contribute to individual risk evaluation and GC prevention.

In this context, finding environmental factors is also important, as not all of the members of the high risk group with OR = 8.4—corresponding to about half of the Japanese population—contract GC. A stratification based on the genotype of GC susceptibility genes may contribute to investigating the environmental factors, as it enables us to concentrate on exploring those that have a critical effect on the high risk group for GC development.

In conclusion, identification of GC susceptibility genes can contribute to GC prevention. To date, aside from 1q22 and 8q24.3, 3 GC-associated loci, chromosome 3q13.31, 5p13.1 and 10q23, have been found [79]. To realize preventive intervention based on genetic risk, additional GC susceptibility genes should be identified in and outside of the loci in order to stratify the population in a more detailed manner.

## Figures and Tables

**Table 1.** Association between GC (gastric cancer) and *MUC1* SNPs (single nucleotide polymorphisms). Ref. = Reference.

SNPs (Major/Minor)	Risk Allele	Odds Ratio (95% CI) and Genotype	*p* Value	Ethnic	Cancer Type	Ref.
rs4072037 (A/G)	A	1.62 (1.32–1.99) # A to G, allelic	4.04 × 10^−6^	Japanese	DGC	[9]
rs4072037 (A/G)	A	1.74 (1.26–2.39) # A to G, allelic	7.82 × 10^−4^	Korean	DGC	[9]
rs4072037 (A/G)	A	0.78 (0.67–0.91) AG to AA	0.031	Korean	All	[30]
rs4072037 (A/G)	A	0.72 (0.62–0.85) G to A, allelic	5.74 × 10^−5^	Chinese	Non-cardia	[11]
rs4072037 (A/G)	A	0.75 (0.65–0.87) G to A, allelic	9.45 × 10^−5^	Chinese	Cardia	[11]
rs4072037 (A/G)	A	0.73 G to A, allelic	1.0 × 10^−4^	Chinese	Non-cardia	[10]
rs4072037 (A/G)	A	1.81 AA to AG + GG	0.031	Chinese	All	[31]
rs2070803 (G/A)	G	0.46 (0.32–0.67) AA + AG to GG	<0.001	Chinese	All	[32]
rs4072037 (G/A)	A	2.20 (1.41–3.44) AA to GG	<0.01	Caucasian	All	[33]
rs4072037 (G/A)	A	0.5 (0.3–0.9) AG to AA	-	Caucasian	Cardia	[34]
rs4072037 (G/A)	A	0.4 (0.2–0.9) AG to AA	-	Caucasian	Non-cardia, Intestinal	[34]

# additive model.

**Table 2.** MUC1 expression in gastric cancer observed by immunohistochemistry.

Study	MUC1 Staining							Correlation to Clinical Information

	Intestinal		Diffuse		Intestinal + Diffuse		Note	
		
	Case No.	(%)	Case No.	(%)	Case No.	(%)		
Ho, *et al*. [38]	-	-	-	-	25/33	(75.8)	-	-

Reis, *et al*. [40]	31/31	(100)	24/24	(100)	-	-	fully glycosylated MUC1	lymphatic invasion *, nodal metastasis *, advanced stage

-	73/90	(81.1)	30/49	(61.2)	-	-	under-glycosylated MUC1	wall penetration, lymphatic invasion *, nodal metastasis, advanced stage

Utsunomiya, *et al*. [39]	(60/68)	(88)	(45/68)	(66)	-	-	fully glycosylated MUC1	worse prognosis *

Lee, *et al*. [41]	37/113	(32.7)	28/159	(17.6)	-	-	-	worse prognosis *

Wang, *et al*. [42]	13/21	(61.9)	11/17	(64.7)	-	-	-	better prognosis

Wang, *et al*. [43]	14/26	(53.8)	30/44	(68.2)	-	-	-	worse prognosis *

Kocer, *et al*. [45]	10/16	(62.5)	13/19	(68.4)	-	-	-	worse prognosis *

Barresi, *et al*. [44]	23/27	(85.2)	3/10	(30)	-	-	-	-

Terada, *et al*. [46]	-	-	3/30	(10)	-	-	signet-ring cell carcinoma	-

* Statistically significant correlation was demonstrated.

**Table 3.** Effect of HP (*Helicobacter pylori)* infection and MUC1 polymorphism on GC risk [60].

Factors	GC Risk		
*MUC1* polymorphism	rs4072037	GG, AG	AA
	tandem-repeat [59]	LL, LS	SS
	splicing variant [9]	2/2, 2/3	3/3

*HP* infection	seronegative	1.00 (reference)	2.46 (1.42–4.27) #
	seropositive	2.30 (1.23–4.31) #	3.95 (2.29–6.79) #

# Odds ratio (95% CI).
